# Spatial Distribution of Reef Fish Species along the Southeast US Atlantic Coast Inferred from Underwater Video Survey Data

**DOI:** 10.1371/journal.pone.0162653

**Published:** 2016-09-21

**Authors:** Nathan M. Bacheler, Zebulon H. Schobernd, David J. Berrane, Christina M. Schobernd, Warren A. Mitchell, Bradford Z. Teer, Kevan C. Gregalis, Dawn M. Glasgow

**Affiliations:** 1 National Marine Fisheries Service, Southeast Fisheries Science Center, 101 Pivers Island Road, Beaufort, North Carolina, 28516, United States of America; 2 Marine Resources Research Institute, South Carolina Department of Natural Resources, 217 Fort Johnson Road, Charleston, South Carolina, 29412, United States of America; University of Sydney, AUSTRALIA

## Abstract

Marine fish abundance and distribution often varies across spatial scales for a variety of reasons, and this variability has significant ecological and management consequences. We quantified the distribution of reef-associated fish species along the southeast United States Atlantic coast using underwater video survey samples (*N* = 4,855 in 2011–2014) to elucidate variability within species across space, depths, and habitats, as well as describe broad-scale patterns in species richness. Thirty-two species were seen at least 10 times on video, and the most commonly observed species were red porgy (*Pagrus pagrus*; 41.4% of videos), gray triggerfish (*Balistes capriscus*; 31.0%), black sea bass (*Centropristis striata*; 29.1%), vermilion snapper (*Rhomboplites aurorubens*; 27.7%), and red snapper (*Lutjanus campechanus*; 22.6%). Using generalized additive models, we found that most species were non-randomly distributed across space, depths, and habitats. Most rare species were observed along the continental shelf break, except for goliath grouper (*Epinephelus itajara*), which was found on the continental shelf in Florida and Georgia. We also observed higher numbers of species in shelf-break habitats from southern North Carolina to Georgia, and fewer in shallower water and at the northern and southern ends of the southeast United States Atlantic coast. Our study provides the first broad-scale description of the spatial distribution of reef fish in the region to be based on fishery-independent data, reinforces the utility of underwater video to survey reef fish, and can help improve the management of reef fish in the SEUS, for example, by improving indices of abundance.

## Introduction

Elucidating the distribution of marine fish species over both time and space is central for their effective management. However, most research on marine fish abundance has historically focused on the causes and consequences of variability in marine fish abundance over time (e.g., [1–2]). But it is now well known that all marine fish populations exhibit spatial structure or variability over a range of spatial scales from small schools to metapopulations [3], and this spatial variability has ecological and management significance [4]. For instance, survival rates often vary across space [5], so understanding the drivers of spatially variable survival can shed light on the mechanisms that ultimately cause variability in population abundance over time.

A major driver of spatial variability in the distribution of marine fish is habitat. Species tend to be found across habitats in a way that allows them to maximize prey consumption and minimize predation threat [6]. Thus, habitat use by marine fish is often a product of interacting density-dependent and density-independent processes [7]. Unfortunately, the efficiency of sampling gears often varies across different habitat types, making it difficult to investigate the relationship between habitat and fish abundance. For instance, American lobsters (*Homarus americanus*) and various temperate reef fish species are more efficiently sampled with traps on sand and mud habitats compared to rocky, hardbottom areas, so even though their densities are much higher in rocky areas, it appears (based on trap-survey data) as though these species have no habitat preferences when in fact they do [8–9]. Similarly, some species avoid trap sampling gears entirely [10]. Using a gear like underwater video that has fewer issues with variable detection across habitat types [9,11] and is more sensitive to the abundance of trap-shy species [10], is critical when making inferences about marine fish habitat use.

Here we describe the distribution of reef-associated fish species using presence-absence data from a large-scale (> 80,000 km^2^) underwater video survey operating annually along the southeast United States Atlantic coast (hereafter, “SEUS”). There were two objectives of our work. Our first objective was to quantify the ways in which the distribution of reef fish species varied (within species) across space, depth, and habitat. Our second objective was to identify where the highest and lowest number of species were seen on videos, to make inferences about patterns of species richness across the SEUS. We hypothesized that reef fish species in the SEUS would be non-randomly distributed across space, depths, and habitats in our study, and that the highest species richness would occur on the outer continental shelf. We expect three applied benefits from addressing these objectives: (1) improving precision and accuracy of indices of abundance due to a better understanding of the spatial range over which these species occur [12], (2) providing a baseline spatial distribution upon which future changes (e.g., shifts in species’ distribution) can be compared [13], and (3) delineating the location of species-diverse “hotspots” that can be used for marine protected area planning [14].

## Materials and Methods

### Ethics statement

Data collection for this study was authorized in a 5-year Scientific Research Permit (that commenced in 2010), issued by the Administrator of Southeast Regional Office of the National Marine Fisheries Service, National Oceanic and Atmospheric Administration, United States Government. This Scientific Research Permit covered all areas and organisms sampled in the study. All research followed the guidelines of the U.S. Government Principles for the Utilization and Care of Vertebrate Animals Used in Testing, Research, and Training (http://grants.nih.gov/grants/olaw/references/phspol.htm#USGovPrinciples).

### Study area

**Fig 1:**
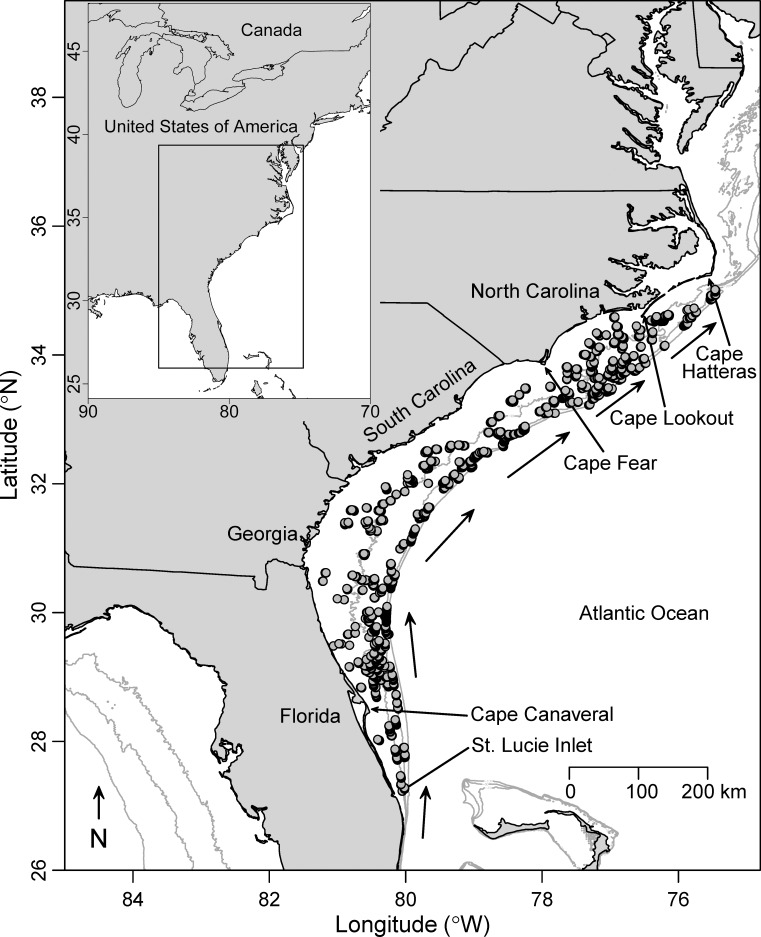
Locations of video samples included in the analyses from the Southeast Reef Fish Survey. Data is from the southeast United States, 2011–2014 (gray points; *N* = 4,855 videos in total). Note that points often overlap. Gray isobaths indicate 30-, 50-, and 100-m depths, and arrows indicate the general path of the Gulf Stream.

Our sampling took place on the continental shelf and upper slope of the SEUS, a large (> 100,000 km^2^) region between Cape Hatteras, North Carolina, and St. Lucie Inlet, Florida (Fig 1). The width of the continental shelf varies from as narrow as 10 km in southern Florida to over 120 km off Georgia. The Gulf Stream is the dominant oceanographic feature in the SEUS and is a warm and powerful northward-flowing ocean current that influences the oceanography and temperature dynamics of the outer continental shelf regions in the SEUS, especially off Florida and North Carolina ([15]; Fig 1).

### Data collection

We used video data from a large-scale fishery-independent survey called the Southeast Reef Fish Survey (SERFS) for all analyses [9]. The SERFS survey has used chevron traps to index reef fish abundance in the region since 1990 (see [16–17] for more details), and since 2011, video cameras have been attached to all traps in a consistent way to provide additional abundance and distribution information on these species [10]. Here we use SERFS video data from 2011–2014 to make inferences about the spatial distribution and habitat use of numerous reef fish species in the SEUS.

The SERFS survey samples hard substrate on the continental shelf and shelf-break in the SEUS, the primary habitat of reef fish in the region. Most of the substrate in the SEUS is sand and mud, but hard substrates occur in patches throughout the region [18]. Hard substrates in the region range from flat limestone pavement, sometimes covered in a thin sand veneer, to high-relief (i.e., 15 m) ledges. A simple random sampling design was used to select stations for sampling each year from a sampling frame of approximately 3,000 stations on or very near hardbottom habitat. In order to increase sampling efficiency, some stations in the sampling frame were sampled opportunistically even though they were not randomly selected for sampling in a given year. Furthermore, samples from newly discovered hardbottom stations were also included in the analyses if hardbottom habitat or reef fish species were present. Sampling occurred from approximately April to October each year on the R/V *Savannah*, R/V *Palmetto*, or NOAA Ship *Pisces*.

High-definition Canon® Vixia HF-S200 video cameras in Gates HF-S21 housings were attached over the mouth of each baited chevron trap deployed in the study, facing away from traps (see [9,16] for a complete description). Briefly, chevron traps were approximately 1.7 m × 1.5 m × 0.6 m in size and shaped like an arrowhead, with a total volume of 0.91 m^3^. Each chevron trap with attached video cameras was baited with 24 menhaden (*Brevoortia* spp.) [9–10]. A second high-definition GoPro® Hero video or Nikon Coolpix S210/S220 still camera was attached over the nose of all traps in order to quantify habitat features in the opposite direction. Only the Canon video cameras over the trap mouth were used for counting fish, and default setting for all cameras were used when recording videos. Traps with attached video cameras were deployed in groups of up to six traps, with no traps being closer than 200 m from any other trap (often 400-m separation between traps) to provide independence among samples [17]. Traps soaked for approximately 90 minutes, but only the first 30 minutes of video were used for counting fish (see below). Video samples were excluded from analysis if they were too dark to read, the camera was out of focus, video files were corrupted, or the traps bounced or moved significantly after deployment.

Videos were read using the MeanCount approach described in Schobernd et al. [19]. Video reading was conducted over a 20-minute interval commencing 10 minutes after the trap landed on the bottom, to allow time for the trap to settle. A total of 41 “snapshots” were then read, spaced systematically every 30 seconds over a 20-minute period. Here, fish taxa present on any of the 41 snapshots were considered present in that sample. We could have increased the frequency of occurrence for all species had we counted fish in all frames (continuously for 20 minutes) instead of 41 frames, but given the large number of videos collected, budget limitations did not allow for more than 41 frames to be read from each video. Also, the MeanCount approach was used over the more commonly used MaxN (i.e., maximum number of individuals of a species seen on a single frame in a video) because MaxN asymptotes with increasing true abundance and MeanCount does not [19].

Due to time constraints, we also could not read videos for all fish species, so we instead limited our counting to the following 107 “priority” species: (1) the fish species listed in the Fish Stock Sustainability Index [20], (2) highly migratory species such as sharks, mackerels, and tunas, and (3) lionfish *Pterois* spp. due to their significance as an invasive species in the SEUS. We note that one downside of our video-reading approach is that we may not document all species present at each site [9,21], so therefore we view our results as a conservative estimate of the distribution of reef fish species in the SEUS.

Characteristics of the water and habitat were estimated for each station sampled. Substrate was estimated visually from both video cameras as the percent of the bottom that consisted of hard, consolidated sediment at least 10 cm in diameter. Current direction was also estimated visually based on the movement of particles in the water in relation to the primary (Canon) video camera field of view, and was classified as “away,” “sideways,” or “towards.” Water clarity was judged to be “low” if substrate could not be seen by the camera, ‘moderate’ if substrate (but not horizon) could be seen, and ‘high’ if the horizon could be seen in the distance. Bottom water temperature (°C) was measured for each group of simultaneously deployed traps using a ‘conductivity–temperature–depth’ cast, while depth (m), latitude (°N), and longitude (°W) were recorded for each trap using vessel sonar and a global positioning system.

### Data analysis

We analyzed presence and absence data because our objective was to describe the spatial distribution of reef-associated fish species in the SEUS. We first summarized the frequency of occurrence and percent frequency of occurrence for all fish species seen on videos over the four-year time series. For species observed on fewer than 10 videos in total, their locations were plotted but no further analyses were conducted due to low sample sizes.

For fish observed on 10 or more videos, we tested for variation in reef fish presence or absence across space, depth, and substrate using generalized additive models (GAMs), a type of nonlinear regression modeling approach. GAMs are similar to generalized linear models except that a component of each linear predictor is a sum of smooth, nonlinear functions of the predictor variables in the model [22]. We developed binomial GAMs that related the presence or absence of each reef fish species to model covariates (i.e., predictor variables). Models were only developed for fish species if they had a frequency of occurrence of at least 10. A major benefit of using a GAM approach is that we can test for significant effects of space, depth, or substrate on reef fish distribution while standardizing for other variables that may also influence our ability to detect reef fish [9]. The downside is that the power to detect non-random distributions across space, depth, or substrate diminished as sample sizes decreased.

A total of six covariates were related to the presence or absence of reef fish in our GAMs.

In addition to the three covariates of interest (i.e., space, depth, and substrate), we also included year, water clarity, and current direction in the models. Year of the sample was included to standardize for any yearly variability in presence or absence of reef fish, while water clarity and current direction were included because each has been shown to influence the detectability of reef fish on video [9,11]. Multicollinearity among covariates was not a problem given that all variance inflation factors were less than 4.0 [23].

The GAM relating the presence or absence of reef fish to covariates was:

$$\eta =g_{1}(lon,lat)+g_{2}(depth)+g_{3}(substrate)+f_{1}(wc)+f_{2}(cd)+year$$ (1)

where η is the probability of presence on a video by a reef fish species, *lon* is the longitude of the sample, *lat* is the latitude of the sample, *depth* is the depth of the sample, *substrate* is the amount of hard bottom present on videos, *wc* is water clarity, *cd* is current direction, *year* is the year of the sample, *g*_1–3_ are nonparametric smoothing functions, and *f*_1–2_ are categorical functions. Latitude and longitude were included as a single, two-dimensional, smoothed covariate (hereafter referred to as “position”) to capture any potential spatial variation in distribution that might vary by more than simply latitude or longitude by themselves, given that the SEUS region is not oriented north-south or east-west. All GAMs were coded and analyzed in R version 3.1.2 [24] using the mgcv library 1.8–4 [25]. The degree of flexibility in the smoothed covariates was determined automatically using the built-in algorithm in the mgcv library. All final GAMs met assumptions of normality and constant variance using diagnostics produced by the “gam.check” function.

The assumption of sample independence was tested by constructing semivariograms that were developed using video data for each of the species analyzed in this study. Semivariograms are commonly used to compare the semivariance (i.e., dissimilarity) of all pairs of samples to the distance between those pairs of samples, and the presence of spatial autocorrelation is apparent when an asymptotic relationship is observed between semivariance and the distance between points. Spatial autocorrelation was not apparent for any of the species in our dataset based on semivariograms (S1 Appendix), likely because most of the video samples were separated by at least 400 m. Thus, there was no indication that the assumption of spatial independence was violated.

We created two types of visualizations from the output of the GAMs. The first visualization was used specifically for the effect of position, since it was a two-dimensional variable. Here, we used a two-dimensional color (heat) plot for each species, showing the likelihood of that species to be seen across space within the SEUS. For each species, the observed presence-absence data from videos was overlaid on top of the plots, and plots were not shown for species where the position variable was non-significant.

For the effects of depth and substrate, we used a second visualization where the one-dimensional GAM fit for each species was overlaid on the raw observed data for each variable. To represent observed data, depth was binned into 10-m bins (e.g., 10–19 m, 20–29 m, etc.) except that 80–110 m was combined into a single bin due to low sample sizes in this depth zone. The maximum depth encountered in the survey was 110 m. Substrate was binned into the following categories that had similar sample sizes: no hardbottom present, 1–4% hardbottom, 5–9% hardbottom, 10–39% hardbottom, and 40–100% hardbottom. The overall percent frequency of occurrence for each species and variable was shown as a horizontal dashed line on these plots. If a predictor variable was insignificant for a particular species in the GAM, the black line representing model fit was removed.

Our last objective was to develop a GAM to identify areas where the highest and lowest number of species were seen on videos in the SEUS. Here, we used the same modeling formulation as Eq 1 above with three differences. First, the response variable was changed to reflect the number of species seen on video from each sample. Second, bottom water temperature (°C) was included as a predictor variable, which was not feasible for the models described earlier due to limited degrees of freedom available for most species. Third, since our response variable was now a count variable, we were unable to use a binomial error distribution. Instead, we compared three alternative error distributions (i.e., Poisson, negative binomial, Tweedie) commonly used for count data, and found that the Poisson distribution outperformed the other distributions based on the pattern of residuals, so it was used here. In addition to plots showing the effect of covariates on the number of species seen on video, we included a two-dimensional heat plot across the SEUS showing the predicted number of species seen on video that was overlaid with the original video observations.

## Results

A total of 4,855 videos were included in our analyses from 2011–2014, ranging from Cape Hatteras, North Carolina, to St. Lucie Inlet, Florida (Fig 1). A total of 32 priority fish species was observed on at least 10 videos over the time series (Table 1). Red porgy (*Pagrus pagrus*) was the most commonly observed species (*N* = 2,010; 41.4% of videos), followed by gray triggerfish (*Balistes capriscus*; *N* = 1,503; 31.0%), black sea bass (*Centropristis striata*; *N* = 1,413; 29.1%), vermilion snapper (*Rhomboplites aurorubens*; *N* = 1,345; 27.7%), and red snapper (*Lutjanus campechanus*; *N* = 1,099; 22.6%; Table 1). Silk snapper (*Lutjanus vivanus*) was the least frequently observed species (*N* = 10; 0.2%) meeting our minimum sample size threshold.

**Table 1:** Sampling information for the 32 most common species seen on video in the Southeast Reef Fish Survey.

Common name	Scientific name	FO	%FO	Latitude (°N) range	Depth (m) range
Red porgy	*Pagrus pagrus*	2010	41.4	27.3–35.0	20–110
Gray triggerfish	*Balistes capriscus*	1503	31.0	27.2–35.0	15–90
Black sea bass	*Centropristis striata*	1413	29.1	27.2–35.0	15–77
Vermilion snapper	*Rhomboplites aurorubens*	1345	27.7	27.2–35.0	16–107
Red snapper	*Lutjanus campechanus*	1099	22.6	27.3–35.0	16–106
Almaco jack	*Seriola rivoliana*	866	17.8	27.2–35.0	15–110
Greater amberjack	*Seriola dumerili*	774	15.9	27.2–35.0	15–103
Scamp	*Mycteroperca phenax*	591	12.2	27.2–34.6	16–103
White grunt	*Haemulon plumierii*	503	10.4	28.0–34.6	16–57
Lionfish	*Pterois* sp.	496	10.2	27.3–35.0	17–103
Gag	*Mycteroperca microlepis*	343	7.1	27.3–35.0	17–94
Gray snapper	*Lutjanus griseus*	288	5.9	27.3–33.6	15–71
Hogfish	*Lachnolaimus maximus*	248	5.1	29.7–34.6	21–86
Banded rudderfish	*Seriola zonata*	225	4.6	27.2–34.0	17–92
Sand tilefish	*Malacanthus plumieri*	128	2.6	29.9–34.1	29–79
Atlantic sharpnose shark	*Rhizoprionodon terraenovae*	95	2.0	28.8–35.0	16–57
Red grouper	*Epinephelus morio*	84	1.7	27.5–34.9	21–92
Lane snapper	*Lutjanus synagris*	71	1.5	27.3–30.4	15–56
Mutton snapper	*Lutjanus analis*	64	1.3	27.2–33.8	19–97
Rock hind	*Epinephelus adscensionis*	58	1.2	30.0–34.1	21–87
Graysby	*Cephalopholis cruentata*	57	1.2	28.2–34.3	23–76
Cobia	*Rachycentron canadum*	51	1.1	27.4–34.6	20–73
Nurse shark	*Ginglymostoma cirratum*	47	1.0	27.5–34.4	15–55
Blueline tilefish	*Caulolatilus microps*	35	0.7	29.7–34.5	63–94
Snowy grouper	*Epinephelus niveatus*	34	0.7	29.7–34.5	50–110
Yellowtail snapper	*Ocyurus chrysurus*	25	0.5	27.2–33.4	16–62
Sandbar shark	*Carcharhinus plumbeus*	22	0.5	27.9–33.7	20–66
Speckled hind	*Epinephelus drummondhayi*	21	0.4	30.0–35.0	49–98
Tiger shark	*Galeocerdo cuvier*	19	0.4	29.0–34.9	16–71
Lesser amberjack	*Seriola fasciata*	16	0.3	29.7–34.5	21–69
Yellowmouth grouper	*Mycteroperca interstitialis*	14	0.3	32.3–33.9	29–81
Silk snapper	*Lutjanus vivanus*	10	0.2	33.4–33.8	38–100

Videos were collected in 2011–2014 (*N* = 4,855 videos in total). FO = frequency of occurrence (i.e., the number of videos in which the species was present); %FO = percent frequency of occurrence (the percent of videos in which the species was seen).

The distribution of most reef fish varied across space (position), depth, and substrate based on the GAMs (Table 2). Non-random distributions were observed for 69% of reef fish species across position, 66% of species across depths, and 72% of species across substrates (Table 2). Generally, non-random distributions were more likely to be observed across position, depths, and substrates for more commonly seen species compared to rarer species. The deviance explained by the species-specific GAMs ranged from 4.2% (tiger shark *Galeocerdo cuvier*) to 75.1% (blueline tilefish *Caulolatilus microps*), being lower for wide-ranging habitat generalists and higher for habitat specialists (Table 2). Half of the 32 GAMs included a significant *year* effect, while twelve (38%) included *cd* (current direction) and seven (22%) included *wc* (water clarity; Table 2).

**Table 2:** Binomial generalized additive model (GAM) results for the significance of *position*, *depth*, and *substrate* in describing the presence or absence of 32 species seen on at least 10 videos collected by the Southeast Reef Fish Survey, 2011–2014.

	*position*	*depth*	*substrate*	*year*	*wc*	*cd*	Dev
Red porgy	27.9***	6.5***	7.6***	1*	2	2***	36.3
Gray triggerfish	24.8***	7.9***	7.5***	1*	2	2***	13.1
Black sea bass	27.3***	3.7***	7.4***	1***	2***	2***	33.3
Vermilion snapper	28.5***	7.2***	8.4***	1	2***	2***	17.0
Red snapper	27.5***	6.1***	5.7***	1*	2	2***	31.3
Almaco jack	24.4***	6.4***	2.2***	1**	2	2***	15.5
Greater amberjack	16.7***	7.0***	4.4*	1***	2*	2***	7.3
Scamp	19.3***	1.6**	8.5***	1	2	2**	25.2
White grunt	28.1***	4.4***	8.7***	1	2*	2***	49.3
Lionfish	21.5***	7.0***	8.4***	1***	2	2	23.2
Gag	21.4***	6.7**	7.2***	1	2	2	12.0
Gray snapper	19.6***	4.8***	6.1***	1	2**	2	30.5
Hogfish	19.1***	2.4**	6.9***	1*	2	2	36.2
Banded rudderfish	15.8***	4.1*	1.0	1**	2*	2	14.0
Sand tilefish	27.0***	7.7***	1.0***	1**	2	2	45.0
Atlantic sharpnose shark	2.0***	5.6	1.0	1*	2*	2***	12.7
Red grouper	15.0***	4.6	2.6*	1*	2	2***	19.8
Lane snapper	12.3**	1.0*	2.5***	1***	2	2	49.8
Mutton snapper	19.7***	8.6*	1.6	1***	2	2	38.1
Rock hind	21.4	7.7	7.4**	1	2	2	43.4
Graysby	20.8*	7.7	3.7***	1	2	2	36.2
Cobia	16.1	1.0	1.0	1*	2	2***	11.2
Nurse shark	14.6	1.0*	1.0	1	2	2	16.2
Blueline tilefish	16.3	1.0*	3.4*	1	2	2	75.1
Snowy grouper	4.4***	1.0***	1.0*	1	2	2	59.3
Yellowtail snapper	9.4	1.0	1.0**	1	2	2	15.8
Sandbar shark	2.7	2.2	1.0	1**	2	2	13.4
Speckled hind	2.0**	5.2***	1.0***	1	2	2	41.4
Tiger shark	2.6	1.0	1.0	1	2	2	4.2
Lesser amberjack	21.1	1.0	4.0	1	2	2	39.4
Yellowmouth grouper	4.4	1.8	3.1***	1	2	2	32.2
Silk snapper	4.5	1.9	1.0	1	2	2	55.8

Year (*year*), water clarity (*wc*), and current direction (*cd*) were included in the GAMs to standardize for site variability in those effects. Degrees of freedom are provided for categorical variables and estimated degrees of freedom are provided for smoothed variables. Dev is the deviance explained by the GAMs. Asterisks denote significance at the following levels (*p*-values)

* < 0.05

** < 0.01

*** < 0.0001.

**Fig 2:**
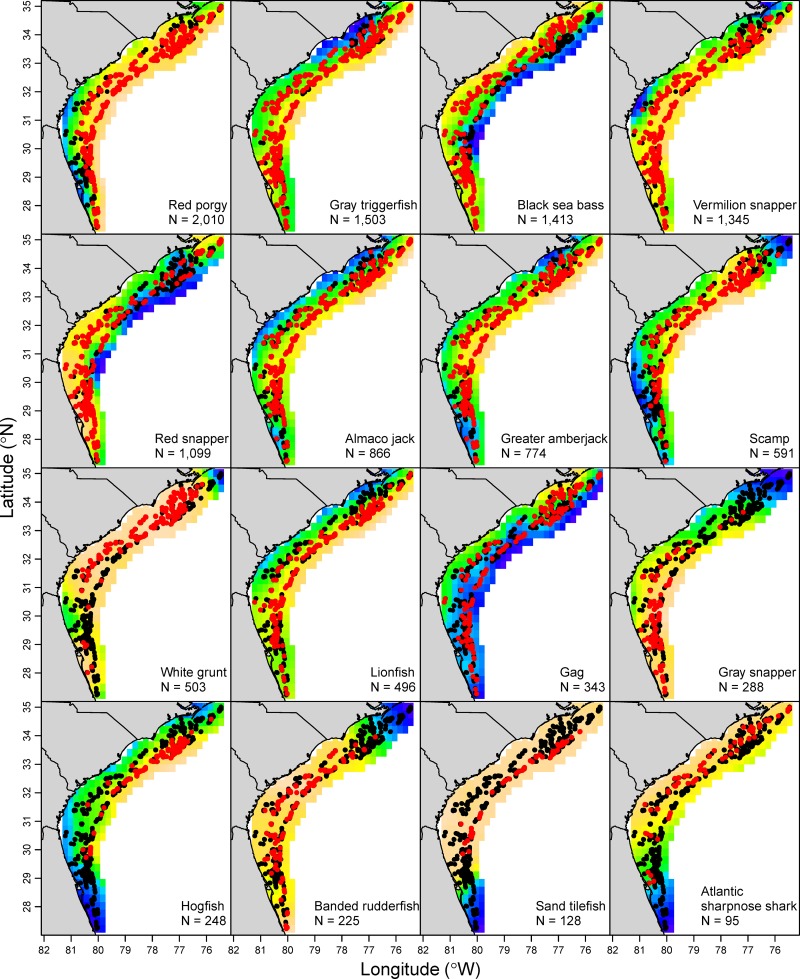
Presence and absence of the 16 most common reef fish species seen on videos. Red points indicate presence and gray points indicate absence from the Southeast Reef Fish Survey, 2011–2014; note that points often overlap. Each background shows the partial effects of position on that species pattern of presence or absence from the generalized additive model; orange is the highest predicted probability of presence and blue is the lowest. The “N” shows the number of videos samples in which the species was present.

**Fig 3:**
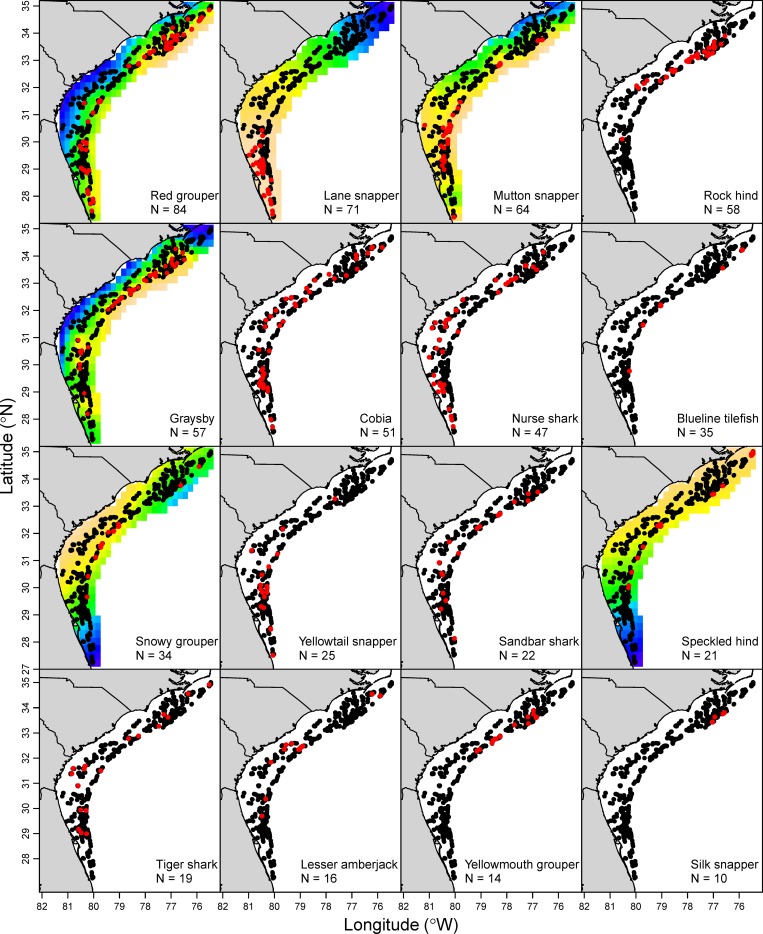
Presence and absence of the next 16 most common reef fish species seen on videos. Red points indicate presence and gray points indicate absence from the Southeast Reef Fish Survey, 2011–2014; note that points often overlap. Each background shows the partial effects of position on that species pattern of presence or absence from the generalized additive model; orange is the highest predicted probability of presence and blue is the lowest, and background heat maps were removed if the position variable was insignificant. The “N” shows the number of videos samples in which the species was present.

Most species were observed over broad areas in the SEUS based on the GAMs, but there were exceptions to this trend (Figs 2 and 3). For instance, white grunt (*Haemulon plumierii*), hogfish (*Lachnolaimus maximus*), and graysby (*Cephalopholis cruentata*) tended to be observed more frequently in the northern SEUS, while gray snapper (*Lutjanus griseus*), lane snapper (*Lutjanus synagris*), and mutton snapper (*Lutjanus analis*) were more frequently observed in the southern SEUS (Figs 2 and 3). While there was a high degree of similarity between the GAM fits and raw observations for most species, there were some exceptions, most notably for snowy grouper (*Epinephelus niveatus*), sand tilefish (*Malacanthus plumieri*), and speckled hind (*Epinephelus drummondhayi*). Moreover, the position variable was not significant for a number of species that appeared to exhibit a non-random spatial distribution (e.g., rock hind *Epinephelus adscensionis*, yellowtail snapper *Ocyurus chrysurus*, yellowmouth grouper *Mycteroperca interstitialis*, silk snapper; Fig 3).

**Fig 4:**
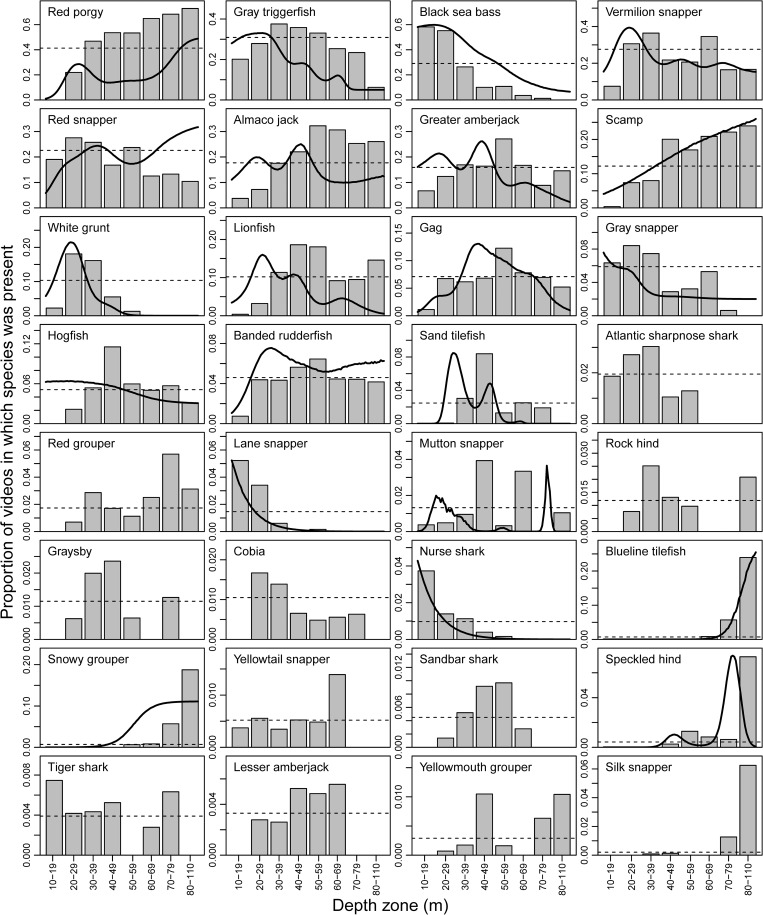
Depth distribution of reef fish species seen on at least 10 videos. Bars show the proportion of videos within each depth zone (m) that the species was seen. The thick black line shows the mean generalized additive model fit for each species, and is absent if it was insignificant. Horizontal dashed lines show the overall proportion of videos in which the species was present. Note different y-axes among panels.

Most species were non-randomly distributed across depths in our survey (Fig 4). Some species tended to be found primarily in shallower water (e.g., black sea bass, red snapper, white grunt, gray snapper, Atlantic sharpnose shark *Rhizoprionodon terraenovae*, lane snapper, nurse shark), some tended to be found in primarily deeper water (e.g., red porgy, scamp *Mycteroperca phenax*, blueline tilefish, snowy grouper, speckled hind, silk snapper), and some appeared to be widely distributed across depths (e.g., gray triggerfish, vermilion snapper, greater amberjack *Seriola dumerili*, gag *Mycteroperca microlepis*, banded rudderfish *Seriola zonata*, tiger shark; Fig 4). Generally, GAM predictions across depths corresponded quite well to the observed data (Fig 4).

**Fig 5:**
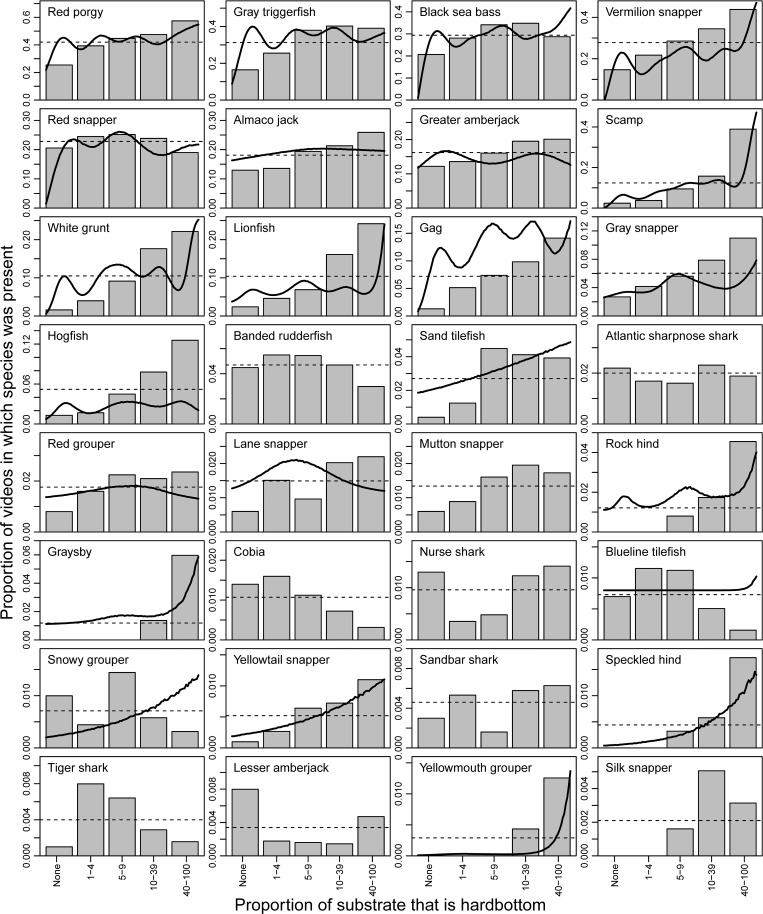
Distribution of reef fish species across substrates that were seen on at least 10 videos. Bars show the proportion of videos within each substrate type that the species was seen. The thick black line shows the mean generalized additive model fit for each species, and is absent if it was insignificant. Horizontal dashed lines show the overall proportion of videos in which the species was present. Note different y-axes among panels.

Variability in the distribution of reef fish across substrate tended to be less apparent than the effects of position or depth, but a majority of species still displayed non-random distributions across substrate (Fig 5). Some species appeared to strongly associate with more continuous hardbottom habitats (e.g., scamp, white grunt, lionfish, gag, hogfish, rock hind, graysby, speckled hind, yellowmouth grouper, silk snapper), while most were found across a variety of substrate categories despite non-random distributions (Fig 5). This included some species like red grouper (*Epinephelus morio*) and snowy grouper that are typically thought to be strongly associated with hard bottom.

**Fig 6:**
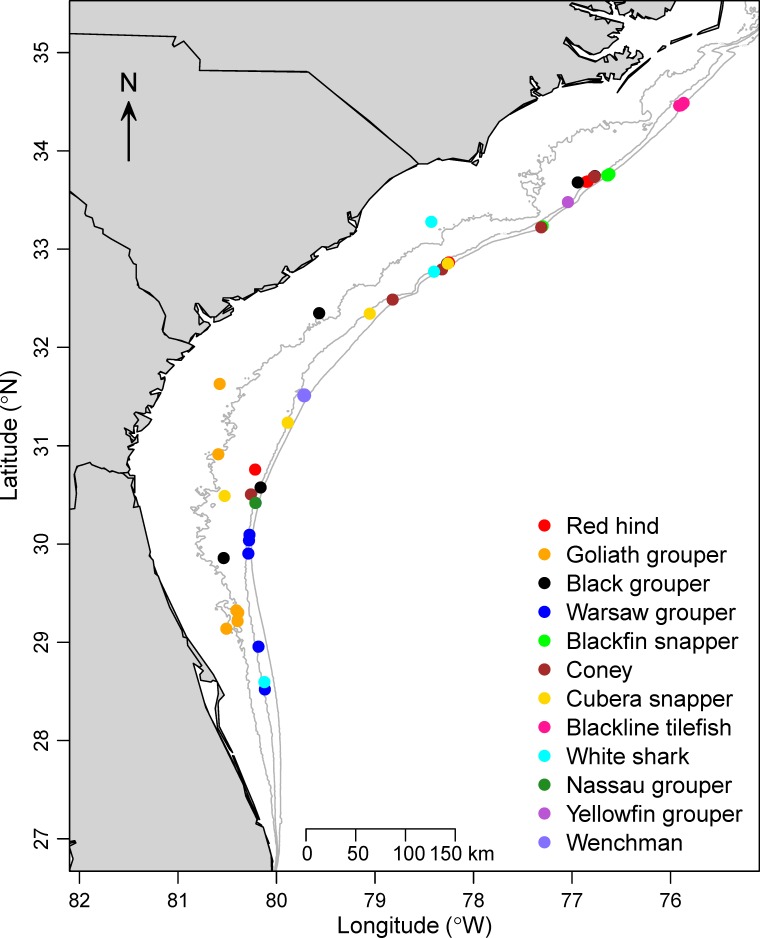
Locations of selected uncommon reef fish species seen on videos. Note that some points overlap. Gray isobaths indicate 30-, 50-, and 100-m depths.

A number of fish species with economic or conservation importance were also seen on videos, but given that they were not observed on at least 10 videos, they were not included in the GAM results above. Some of the more notable rare species observed on video were goliath grouper (*Epinephelus itajara*), warsaw grouper (*Epinephelus nigritus*), cubera snapper (*Lutjanus cyanopterus*), Nassau grouper (*Epinephelus striatus*), and white shark (*Carcharodon carcharias*; Fig 6). Observations of these species were spread out in the SEUS, but tended to occur along the continental shelf break, with the exception of goliath groupers that were found most frequently on the continental shelf off Florida and Georgia (Fig 6).

**Fig 7:**
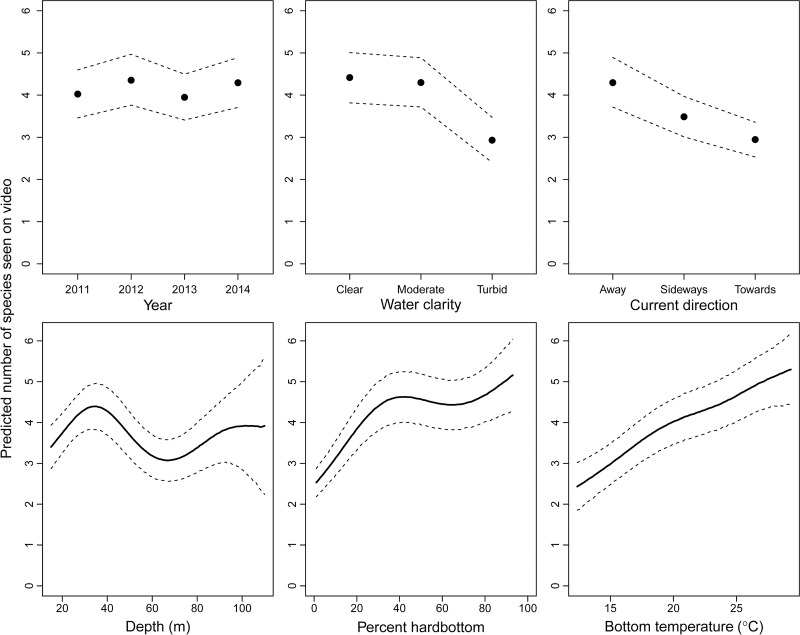
Relationships between the predicted number of priority fish species seen on video and predictor variables. The generalized additive model was built using Southeast Reef Fish Survey video data, 2011–2014. Points (for categorical variables) or solid lines (for smoothed continuous variables) show the mean predicted number of species seen and the dashed lines show 95% confidence intervals.

**Fig 8:**
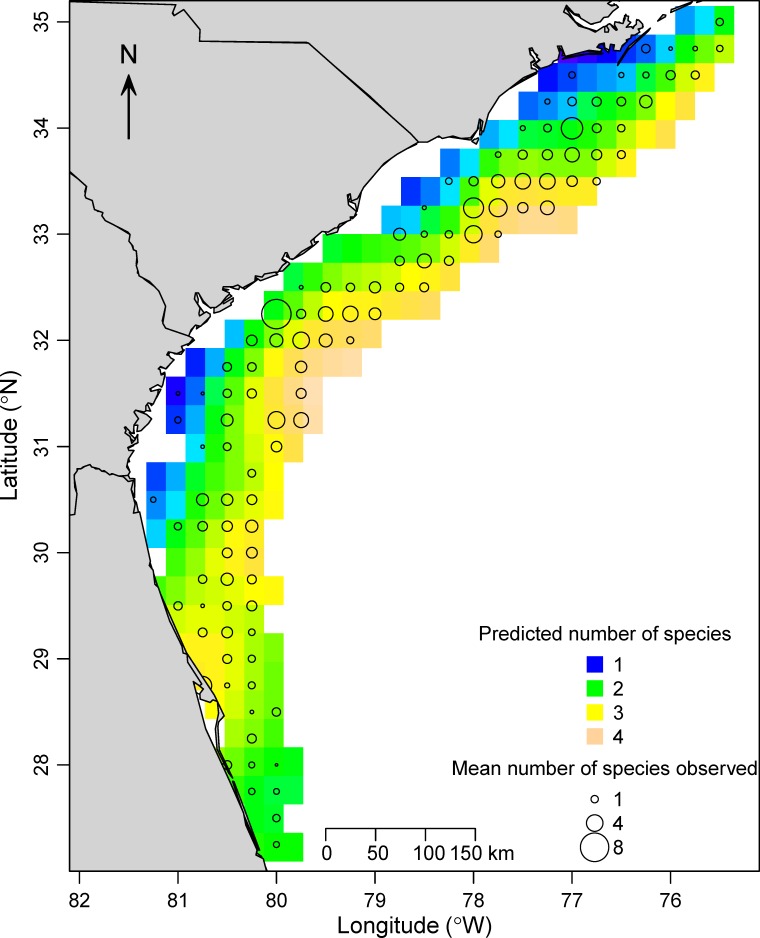
Observed and predicted number of priority fish species seen on video. Spatial predictions for the number of priority fish species on video were made at average values of all other predictor variables in the generalized additive model, and are shown from orange (indicating many species predicted) to blue (indicating low number of species predicted). Open circles show the mean observed number of species on video in 2011–2014 for each 0.25 × 0.25 degree cell.

The GAM model for the number of species observed on video included all predictor variables in the final model and explained 24% of the deviance. Fewer species were seen when water clarity (*P* < 0.001) was low and the current direction (*P* < 0.001) was towards the camera (Fig 7). The number of species observed on video was also variable across years (*P* < 0.001), bimodally related to depth (*P* < 0.001), and positively related to substrate (*P* < 0.001) and bottom water temperature (*P* < 0.001; Fig 7). The mean number of fish species observed on video corresponded well to the spatial predictions from the GAM model (Fig 8). Generally, higher numbers of species were observed on videos in deeper water from southern North Carolina into Georgia, and fewer were observed in shallower water and at the two ends of the SEUS (northern North Carolina and southern Florida; Fig 8).

## Discussion

Based on generalized additive models, reef fish in the SEUS were often found to be non-randomly distributed across space (position), depths, and habitats, especially for those species with larger sample sizes. In nearly all cases, model predictions matched our observations closely. Moreover, models predicted a high degree of variability in species richness across the SEUS, with highest species richness in shelf-break habitats off southern North Carolina through northern Georgia, and lower species richness inshore and at the northern and southern ends of the SEUS. These results can be used to improve the development of reef fish indices of abundance [12], provide baseline spatial distributions upon which future changes can be compared, and develop and refine marine protected area planning [14].

Fish are well known to be patchily distributed at a variety of spatial scales [4]. At the smallest scales, fish school together for a variety of reasons such as limiting predation risk [26], but spatial variability at larger scales can be due to environmental forcing, habitat selection, and the spatial distribution of competitors and predators [7,27]. In our study, reef fish were often non-randomly distributed across space, depths, and habitats, despite the fact that most were widely distributed in the region. The non-random distribution of reef fish in the SEUS was likely due to a variety of interacting processes including schooling behavior, the spatial distribution of hardbottom habitats with which they associate [28], broad variability in environmental conditions such as water temperature [29], and spatially variable patterns of fishing harvest [30].

That the distribution of reef fish was often non-randomly distributed across space, depths, and habitats suggests that there is a high degree of specificity in the ways reef fish use space in the SEUS. It is probably not surprising that most reef fish in the SEUS are non-randomly distributed across space given that the region is large (~1000 km from Cape Hatteras, North Carolina, to St. Lucie Inlet, Florida) and the oceanography of the region is responsible for a high degree of variability in environmental conditions [15]. Depth has also been shown to be a major structuring force for reef fish assemblages in the SEUS [31], likely due to strong depth-specific variability in winter water temperatures and the thermal tolerances of species [32]. There was also considerable variability in the ways in which reef fish used habitat in our study, but most species tended to be more closely associated with hardbottom than sites lacking hardbottom. Many reef fish in the SEUS strongly associate with hardbottom reefs, while others stray off reefs and can periodically be found on adjacent sand and mud substrates [9,18,33]. Our habitat results are conservative because the video samples on sand and mud were not randomly selected, but instead often occurred very close to, and within a short swim of, hardbottom habitat, which was the intended location of trap deployments. If sand and mud habitats far away from hardbottom habitat were sampled in our study, we undoubtedly would have found a much stronger association of fish in our study with hardbottom habitat.

Broad summaries of presence-absence data like those herein can be used to improve the accuracy and precision of indices of abundance. Catch or count data from fishery-independent surveys are often used to provide a relative measure of population abundance (i.e., index of abundance) in fisheries stock assessments [34]. The presence of zero catches, however, is a challenge because of computational issues that arise during the catch-effort standardization process (e.g., log transformations [12]). Moreover, a higher than expected number of zero catches (i.e., zero inflation) can be difficult to properly account for in standardization models [35]. Broad-scale presence-absence data like we provide justifies the removal of samples in areas where the species of interest is rare or absent, which reduces zero inflation and improves the accuracy and precision of indices of abundance. The broad-scale distributions of reef fish from our study can also be used as a baseline upon which future changes in distribution (e.g., changes in species’ distribution) can be compared.

We also used presence-absence data across all priority reef fish species seen on video to make inferences about the broad-scale patterns of species richness in the SEUS. The highest predicted number of species occurred in deep areas off southern North Carolina, South Carolina, and Georgia, while the lowest number of species occurred in shallow water and at the northern and southern ends of the SEUS. These results correspond closely to inferences made in the region using chevron traps over a three-decade time span [29], despite the known differences in the sampling efficiency of underwater video compared to chevron traps [10]. They also correspond to the findings of Whitfield et al. [32], who showed that more (and mostly tropical) fish species tended to occur in deeper waters off southern North Carolina due to warmer wintertime bottom temperatures. Our results have implications for the implementation of marine protected areas, which are typically sited in areas with high species richness (i.e., biological hotspots). Currently, 5 of the 8 marine protected areas designed to protect reef fishes in the SEUS are located in areas we have identified as having high species richness [36].

Underwater video is becoming a primary sampling gear for monitoring reef fish species around the world. Video has gained popularity because it is non-extractive, less selective than traditional fish sampling gears such as trawls or hooks, and can also provide behavioral and habitat information that is often unavailable when using other gears [10,37–38]. In our study, video was able to provide presence-absence data for a variety of economically important priority species, but frequency of occurrence should be considered a minimum estimate because video does not always document all species present at a site due to incomplete detection [9,11]. Reading the entire 20 minutes of underwater video (or more) would likely have increased the frequency of occurrence of reef fish species [21], but would have been more costly. Using bait also likely resulted in higher number of species seen compared to unbaited video [39].

The primary drawback of our study was that a number of species included in the analysis had low sample sizes, resulting in some uncertainty about the distributions of these species in the SEUS. For instance, blueline tilefish were observed on 35 videos in 5 main areas in the SEUS, but their latitudinal distribution would likely be coast-wide if we were able to sample consistently in the depth of water (i.e., 80–200 m) and habitat where blueline tilefish are concentrated. For other species, we likely sampled the core of their distribution, but their frequency of occurrence was low simply due to the fact that they are relatively rare in the SEUS (e.g., yellowmouth grouper). In these instances, our GAMs may have been compromised due to low power.

Our study provides the first broad-scale understanding of the distribution of reef fish across space, depths, and habitats using survey data in the SEUS. Reef fish displayed a high degree of specificity in their distribution, and our modeling results corresponded well to our observations. The distribution of reef fish species varied greatly across the depths, space, and habitats sampled in our study, suggesting these variables may influence reef fish populations elsewhere. Underwater video was a uniquely well-suited methodological approach in our study because the frequency of occurrence of reef fish was generally much higher than would be expected from other gears like traps [10], and we were able to gather important habitat information that is generally unavailable using other survey methods. These results will improve calculations of indices of abundance and, thus, reef fish stock assessments in the SEUS. Moreover, they provide baseline distributions that can be used to compare if changes occur in the future due to species’ distribution shifts, as well as help inform marine protected area siting.

## Supporting Information

S1 Appendix Semivariograms for each reef fish species examined in this study. Semivariograms were created using the same video data that was used in the generalized additive models, and show how similar pairs of samples were at various distances of separation.
(PDF)
