# Supplementary material for: Spatial Distribution of Reef Fish Species along the Southeast US Atlantic Coast Inferred from Underwater Video Survey Data
Source: PLoS One. 2016 Sep 21;11(9):e0162653. doi: 10.1371/journal.pone.0162653 (PMC5031447; doi:10.1371/journal.pone.0162653)

S1 Appendix. Semivariograms for each reef fish species examined in this study. Semivariograms were created using the same video data that was used in the generalized additive models, and show how similar pairs of samples were at various distances of separation.

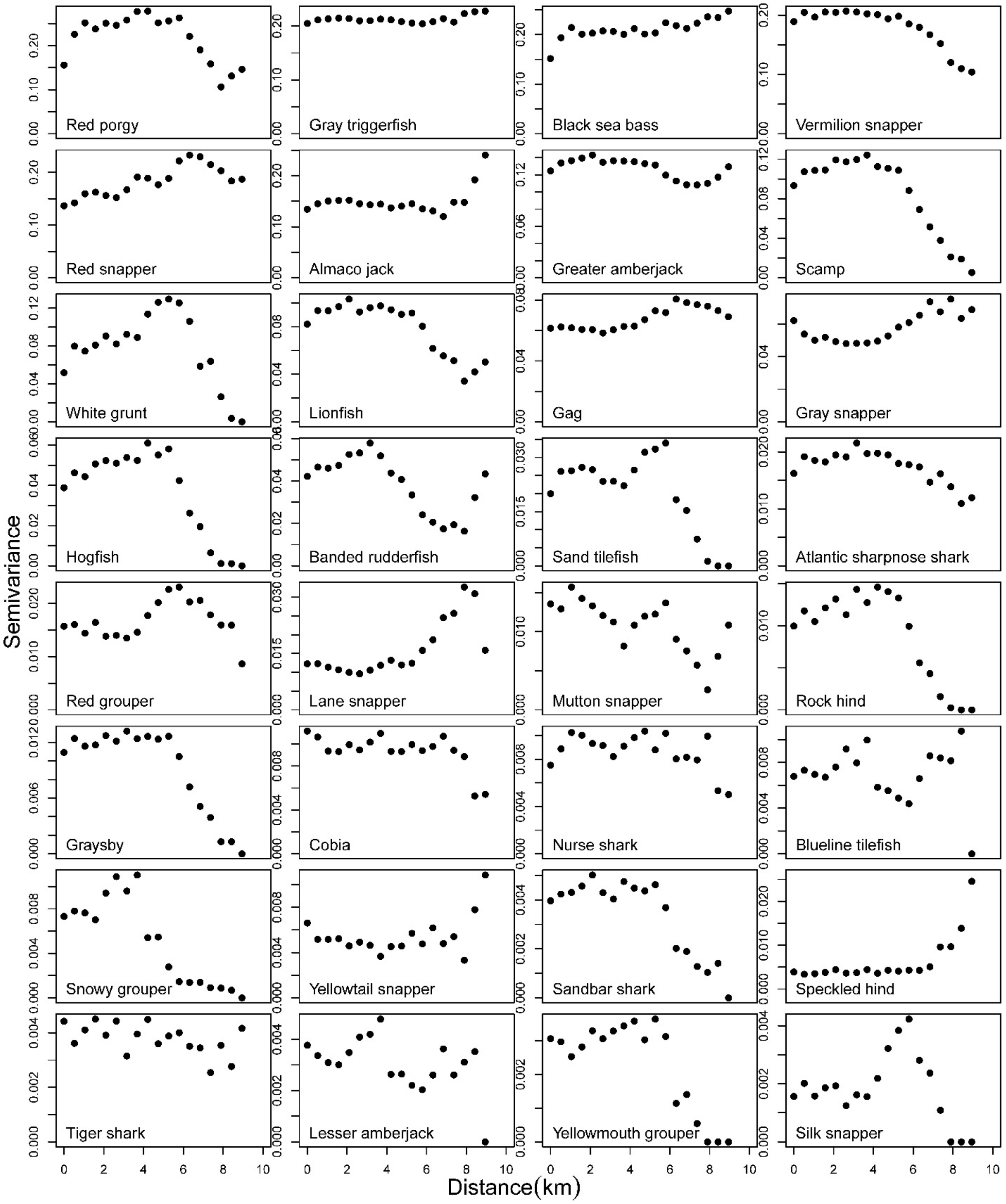

Supplement: S1 Appendix — Semivariograms were created using the same video data that was used in the generalized additive models, and show how similar pairs of samples were at various distances of separation. (PDF) [file pone.0162653.s001.pdf]
